# Arginine Methyltransferases Are Regulated by Epstein-Barr Virus in B Cells and Are Differentially Expressed in Hodgkin’s Lymphoma 

**DOI:** 10.3390/pathogens1010052

**Published:** 2012-09-19

**Authors:** Sarah Leonard, Naheema Gordon, Nikki Smith, Martin Rowe, Paul G. Murray, Ciarán B. Woodman

**Affiliations:** School of Cancer Sciences, College of Medical and Dental Sciences, University of Birmingham, Edgbaston, Birmingham, B15 2TT, UK; E-Mails: s.m.leonard@bham.ac.uk (S.L.); n.gordon@bham.ac.uk (N.G.); smithny@adf.bham.ac.uk (N.S.); rowem@adf.bham.ac.uk (M.R.); murraypg@adf.bham.ac.uk (P.G.M.)

**Keywords:** protein arginine methyltransferases, Epstein-Barr virus, LMP1, Hodgkin’s lymphoma, PRMT1, epigenetics

## Abstract

Although there is increasing evidence that aberrant expression of those enzymes which control protein arginine methylation contribute to carcinogenesis, their de-regulation by oncogenic viruses in primary cells has yet to be reported. We first show that the protein arginine methyltransferases, CARM1, PRMT1 and PRMT5 are strongly expressed in Hodgkin Reed-Sternberg (HRS) cells, and up-regulated in Hodgkin's lymphoma (HL) cell lines. Given that Epstein-Barr virus (EBV) can be detected in approximately 50% of primary HL, we next examined how EBV infection of germinal centre (GC) B cells, the presumptive precursors of HRS cells, modulated the expression of these proteins. EBV infection of GC B cells was followed by the up-regulation of CARM1, PRMT1 and PRMT5, and by the down-regulation of the arginine deiminase, PADI4. Latent membrane protein 1 (LMP1), the major EBV transforming gene was shown to induce PRMT1 in GC B cells and in a stably transfected B cell line. The recent development of compounds which inhibit PRMT-mediated reactions provides a compelling case for continuing to dissect the contribution of virus induced changes in these proteins to lymphomagenesis.

## 1. Introduction

Protein arginine methylation is a post-translational modification which involves the addition of a methyl group to one or two terminal nitrogen atoms on arginine residues. In mammals, there are nine protein arginine methyltransferases (PRMT1-3, CARM1, PRMT5-9,) which catalyse arginine methylation [1]. The peptidylarginine deiminases (PADI), a family of five Ca^2+^ dependent enzymes, effectively act as arginine demethylases by catalysing the conversion of an arginine residue to a citrulline residue [2,3]. In addition to being involved in a number of cellular processes, including DNA repair, RNA transcription, signal transduction and protein compartmentalization, arginine methylation is also important for the replication of viruses and the transcriptional activation and stabilisation of viral proteins [1]. PRMT1 regulates replication of herpes simplex virus; is required for the efficient production of adenovirus type-5; and modulates Kaposi sarcoma-associated herpesvirus gene expression during its life cycle [4,5,6]. PRMT1 and PRMT5 binding to the Epstein Barr virus (EBV) protein EBNA1 is important for the replication and mitotic segregation of viral genomes [7]. Arginine methylation of another EBV oncoprotein, EBNA2, is necessary for its efficient association with DNA bound transcription factors and with other viral promoters [8]. PRMT1. CARM1 enhances transcriptional activation of the HTLV-1 encoded oncoprotein, Tax, and PRMT6 increases the stability of the human immunodeficiency virus type 1 transactivator protein, Tat [9,10]. 

While these examples illustrate how viruses can exploit the cell’s arginine methylation machinery, virus-induced de-regulation of the PRMT has been less extensively investigated. Hepatitis C virus and Human Papilloma Virus E6 protein have been shown to down-regulate arginine methyltransferase activity in transformed cells [11,12]. However, virus induced de-regulation of these proteins in primary cells has not been reported. Here, we investigate how EBV and its major transforming gene, encoding latent membrane protein 1 (LMP1) which can be detected in approximately 50% of cases of primary Hodgkin's lymphoma (HL), modulate the expression of proteins which regulate arginine methylation in germinal centre (GC) B cells, the presumptive progenitor cells of HL [13]. We focus on three PRMT (PRMT1, PRMT5, and CARM1) and one deiminase (PADI4) which were found to be differentially expressed on gene expression profiling of EBV infected GC B cells. PRMT1 contributes 85% of all cellular PRMT activity and co-activates with CARM1, NF-Kappa B dependent gene expression, a pathway which is constitutively activated in many lymphomas including HL [14,15,16]. PRMT5 has been shown to be over-expressed in BL cell lines; to suppress the retinoblastoma family of tumour suppressors in leukaemia and lymphoma cells; and to mediate CCND1-dependent neoplastic growth in a mouse lymphoma model [17,18]. Ectopic expression of the deaminase, PADI4, has been shown to inhibit the growth of transformed B cells [19].

## 2. Results and Discussion

### 2.1. Protein Arginine Methyltransferases Are Differentially Expressed in Primary HL

**Figure 1:**
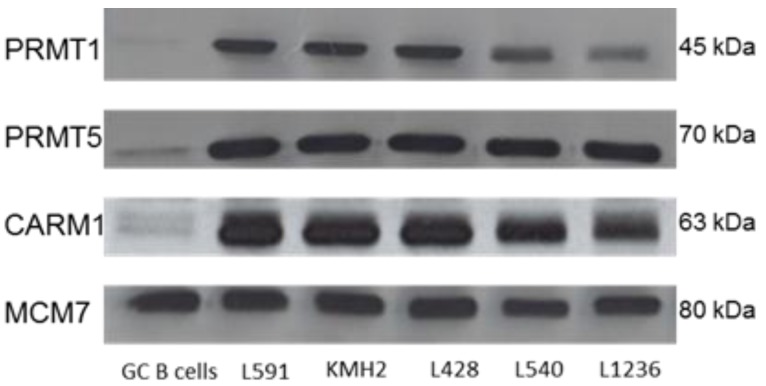
PRMT1, PRMT5 and CARM1 expression in five Hodgkin's lymphoma (HL) cell lines. Western blot showing PRMT expression in HL cell lines compared to GC B cells. MCM7 was used as a loading control.

**Figure 2:**
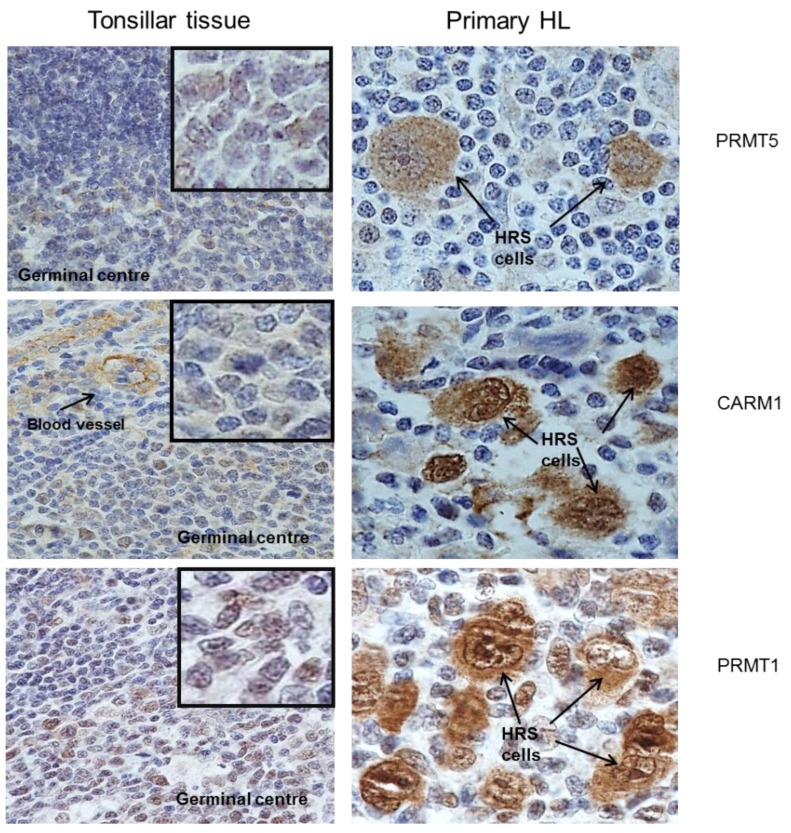
Expression of PRMT in primary HL: Hodgkin Reed-Sternberg (HRS) cellswere strongly positive for PRMT1, PRMT5 and CARM1 when compared to surrounding lymphocytes and tonsillar tissue.

We have previously shown using gene expression profiling, up-regulation of PRMT1, PRMT5 and CARM1 in one or more HL cell lines [20,21]. We were able to confirm increased protein expression of these PRMT in HL cell lines (Figure 1). We also measured using immunohistochemistry, the expression of CARM1, PRMT1 and PRMT5 in 77 cases of primary HL (17 paediatric and 60 adult). These proteins were found to be strongly expressed in HRS cells when compared with surrounding lymphocytes and with normal tonsillar tissue (Figure 2). Consistent with reports describing the distribution of CARM1 and PRMT1 expression in different cell types [1,22,23], there was evidence of both nuclear and cytoplasmic staining for CARM1 and PRMT1 in the majority of patients with HL (Table 1). For PRMT5, staining was predominantly cytoplasmic as has been described elsewhere [24,25] (Table 1). Although the intensity of staining did not vary significantly with histological subtype or age at diagnosis (Supplementary Table 1 and Table 2), the proportion of cases with strong nuclear staining for PRMT1 was greater in those who tested positive for EBV (X^2^_3df_ = 11.0; p = 0.005); CARM1 or PRMT5 expression did not vary significantly with EBV status (Table 2). 

**Table 1:** Immunohistochemical staining of 77 cases of HL for PRMT1, PRMT5 and CARM1. Both nuclear and cytoplasmic PRMT expression was recorded as weak when staining of HRS cells was less than that observed in the surrounding lymphocytes; moderate when staining of HRS cells was as strong as that observed in the surrounding lymphocytes; and strong when staining of HRS cells was greater than that in the surrounding lymphocytes.

Immunohistochemical staining of 77 cases of Hodgkin’s Lymphoma for PRMT1, CARM1 and PRMT5							
Gene		Intensity of Cytoplasmic Staining					
PRMT1 *	intensity of nuclear staining		strong	moderate	weak	negative	Total
		strong	22 (12)	10 (1)	3	8	43
		moderate	0	25 (11)	3	4	32
		weak	0	0	1	0	1
		negative	0	0	0	0	0
CARM1 **	intensity of nuclear staining	strong	26	6	0	0	32
		moderate	11	14	1	0	26
		weak	4	3	4	0	11
		negative	0	1	2	1	4
PRMT5 *	intensity of nuclear staining	strong	4 [2]	0	0	0	4
		moderate	2 [2]	10 [5]	0	0	12
		weak	7	5	1	0	13
		negative	25	21	0	1	47

* 1 and ** 4 cases were not evaluable. Numbers and parenthesis refer to those cases which cytoplasmic staining was occasional. Numbers in square brackets refer to those cases in which nuclear staining was occasional.

**Table 2:** Influence of EBV status: comparison of immunohistochemical staining for PRMT1, CARM1 and PRMT5 in nodular sclerosis and mixed cellularity HL. Both nuclear and cytoplasmic PRMT expression was recorded as weak when staining of HRS cells was less than that observed in the surrounding lymphocytes; moderate when staining of HRS cells was as strong as that observed in the surrounding lymphocytes; and strong when staining of HRS cells was greater than that in the surrounding lymphocytes.

Influence of EBV Status: Comparison of Immunohistochemical Staining for PRMT1, CARM1 and PRMT5 in EBV Positive (n = 27) and EBV Negative (n = 50)							
intensity of nuclear staining		PRMT1		CARM1		PRMT5	
		EBV negative	EBV positive	EBV negative	EBV positive	EBV negative	EBV positive
	strong	21	22	24	9	3	2
	moderate	27	5	16	9	7	4
	weak	1	0	5	6	8	5
	negative	0	0	3	1	31	16
		X^2^_3df_ = 11; p = 0.005		X^2^_3df_ = 2.9; p = 0.41		X^2^_3df_ = 0.1; p = 0.99	
intensity of cytoplasmic staining	strong	12	10	26	16	25	13
	moderate	25	10	14	9	23	13
	weak	6	1	7	0	0	1
	negative	6	6	1	0	1	0
		X^2^_3df_ = 4.2; p = 0.24		X^2^_3df_ = 4.4; p = 0.06		X^2^_3df_ = 3.4; p = 0.3	

* 1 and ** 4 cases were not evaluable. Numbers and parenthesis refer to those cases which cytoplasmic staining was occasional. Numbers in square brackets refer to those cases in which nuclear staining was occasional.

### 2.2. EBV Infection Modulates the Expression of the Protein Arginine Methyltransferases

**Figure 3:**
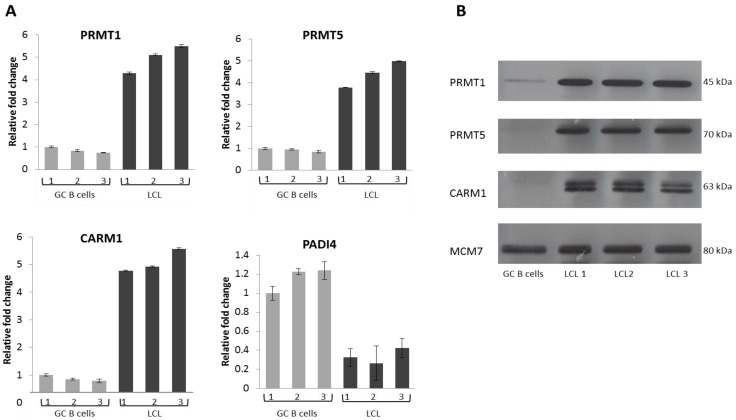
(**A and B**) PRMT1, PRMT5, CARM1 and PADI4 expression in GC B cells and EBV infected GC B cells. (**A**) Q-RT PCR showing PRMT and PADI4 expression. Grey bars represent expression in GC B cells isolated from three different patients (1,2,3) and black bars that in each of the corresponding EBV infected GC B cells. The GC B cells with the highest expression of the gene in question served as the reference sample. Assays were carried out in triplicate and results are presented as 2^−∆∆CT^ values; (**B**) Western blot showing PRMT expression in GC B cells and in corresponding EBV infected GC B cells; MCM7 was used as a loading control.

**Figure 4:**
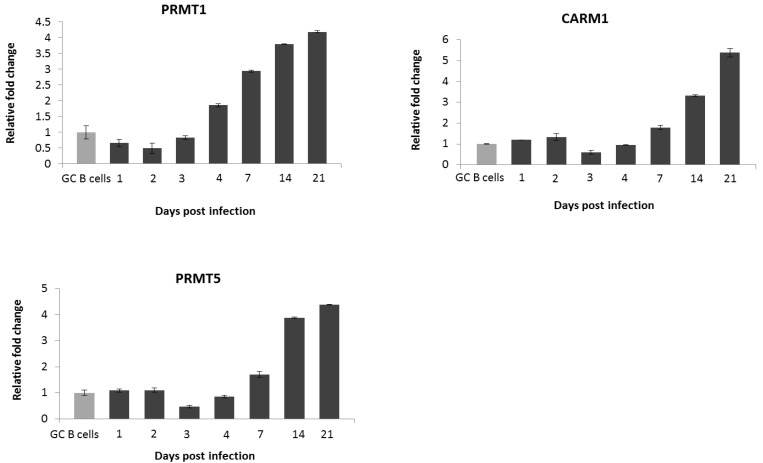
Kinetics of PRMT1, PRMT5 and CARM1 expression in GC B cells and in LCLs. Q RT-PCR showing changes in PRMT expression in GC B cells infected with EBV compared to GC B cells. Assays were carried out in triplicate and results are presented as 2^−∆∆CT^ values. Experiments were performed on the three LCLs and representative results for one LCL are shown.

As EBV is believed to contribute to the pathogenesis of HL, we next investigated whether this oncogenic virus modulates the expression of these proteins in germinal centre (GC) B cells, the presumptive progenitor cells of HL. We first examined the expression of these proteins in three LCL derived from GC B cells isolated from different donors. Compared with their expression in GC B cells, PRMT1, PRMT5 and CARM1 were up-regulated at the RNA (Figure 3A) and protein level (Figure 3B) in all three LCL. PADI4 was substantially decreased at the RNA level (Figure 3A), but its protein could not be examined because no western blotting antibody was available. Using RNA collected from earlier time-points we found that PRMT1 was up-regulated within 96 hours of EBV infection whilst up-regulation of PRMT5 and CARM1 was delayed for 7 days (Figure 4). 

### 2.3. PRMT1 is Up-Regulated in B Cells by the EBV Oncogene, LMP1

**Figure 5:**
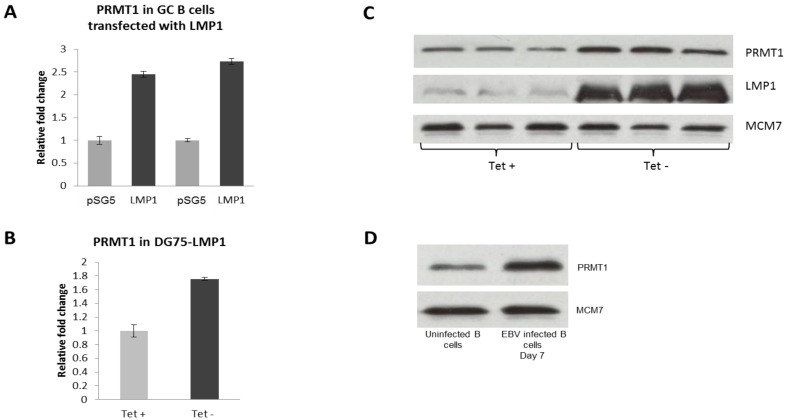
(**A–D**): LMP1 up-regulates PRMT1. (**A**) Q RT-PCR showing up-regulation of PRMT1 following transfection of GC B cells with LMP1. Assays were performed in triplicate and the results are presented as 2^−∆∆CT^ values compared to the vector control, pSG5; (**B and C**) LMP1-induced up-regulation of PRMT1 at the RNA and protein level using a tetracycline-inducible expression system. Tet + refers to the cells grown in the presence of tetracycline and Tet- to those grown without. Protein assays were performed in triplicate. MCM7 was used as a loading control; (**D**) Western blotting showing the increase in PRMT1 expression 7 days post infection of naive B cells with EBV. MCM7 was used as a loading control.

Given that we and others have shown that the major EBV transforming gene in HL, latent membrane protein 1 (LMP1), is usually first detected 72–96 hours following EBV infection [21], we next investigated whether this oncogene modulated the expression of PRMT1, PRMT5 and CARM1 in LMP1-transfected CD10^+ve^ GC B cells. Towards this end, GC B cells isolated from two tonsils removed from different patients were transfected with either an LMP1-expressing pSG5-LMP1 expression vector or with a pSG5 vector control as previously described [21]. Q RT-PCR confirmed the up-regulation of PRMT1 in RNA isolated 24 hours following transfection with LMP1 from both preparations of GC B cells (Figure 5A). However, we found no evidence to suggest that LMP1 regulates the expression of either PRMT5 or CARM1 in GC B cells. We confirmed LMP1-induced up-regulation of PRMT1 at the RNA and protein level in the EBV negative BL cell line DG75 using an inducible expression system in which removal of tetracycline is followed by induction of LMP1 expression (Figure 5B and 5C) [26]. PRMT1 was also shown to be up-regulated at the protein level in naive B cells 7 days following EBV infection (Figure 5D). However, we found no evidence to suggest that LMP2A or EBNA1 modulates the expression of any of the enzymes under consideration in GC B cells (data not shown). 

## 3. Experimental Section

### 3.1. Isolation and Infection of Tonsillar GC B Cells

Tonsillar tissue was obtained from the Children’s Hospital Birmingham following informed consent (reference number for ethical approval 06/Q2702/50). Mononuclear cells were isolated by Ficoll-Isopaque centrifugation and CD10^+^ GC B cells by magnetic separation on LS columns (Miltenyi Biotec, Germany) using α-CD10-Phycoerythrin (PE) (eBioscience, UK) and α-PE microbeads (Miltenyi Biotec). Wild-type 2,089 EBV particles were produced from 293 cells carrying a recombinant B95.8 EBV genome (kindly provided by Dr. Claire Shannon-Lowe) and virus copy number was measured using a BALF5 quantitative PCR (Q-PCR) assay. GC B cells (2 × 10^6^) were infected overnight on a fibroblast feeder layer with 2,089 EBV at a multiplicity of infection of 50.

### 3.2. Maintenance of Cell Lines

GC B cell derived LCLs were established and maintained for six weeks at 37 °C in RPMI 1,640 medium (Sigma-Aldrich, Missouri USA) supplemented with 10% foetal calf serum (FCS) and 1% penicillin/streptomycin (Invitrogen, CA, USA) (20). LMP1 inducible DG75 cells were maintained in RPMI 1640, 10% FCS, 1% penicillin/streptomycin, 1.5 mg/mL G418, 0.5 mg/mL hygromycin (Sigma-Aldrich) and 1 µg/mL tetracycline; whereas cells cultured with tetracycline did not express LMP1, those grown in the absence of tetracycline expressed this viral oncogene (26). 

### 3.3. Quantitative Reverse Transcriptase-Polymerase Chain Reaction

Total RNA was extracted using RNeasy Mini kit (Qiagen, Germany). cDNA was generated using the Superscript III First-strand synthesis system (Invitrogen) with random primer (Promega, UK). Q-PCR assays were prepared in a final volume of 25 μL which contained 1 μL cDNA, TaqMan universal PCR mastermix (Applied Biosystems, CA, USA), *B2M *house-keeping assay (Applied Biosystems) and Taqman assay for the target genes, *PRMT1* Hs01587651_g1, *PRMT5* Hs01047356_m1, *CARM1* Hs00406354_m1 and *PADI4 *Hs00202612_m1 (Applied Biosystems). Q-PCR assays were performed in triplicate using an ABI Prism 7,700 sequence detection system (Applied Biosystems). The 2^−ΔΔ^ CT method was used to quantify expression relative to the housekeeping control. 

### 3.4. Western Blotting

Cells (1 × 10^7^) were lysed in 100 µL RadioImmuno Precipitation Assay (RIPA) buffer (50 mM Tris-HCl pH 8, 150 mM NaCl, 1% Triton X-100, 0.5% sodium deoxycholate, 0.1% sodium dodecyl sulfate (SDS), 1 mM sodium vanadate and protease inhibitor cocktail (Promega)). Protein was denatured by heating to 90 °C in SDS buffer, run on a–polyacrylamide gel before being transferred to BioTrace NT membrane (VWR International, USA), and then incubated overnight with primary antibody diluted in 5% (w/v) milk. Antibodies used were: PRMT1 mouse monoclonal antibody (Sc-59648, Santa Cruz, California, USA) at 1:2,000 dilution; PRMT5 mouse monoclonal antibody (Ab-12191, Abcam, Cambridge, UK) at 1:2,000 dilution; CARM1 mouse monoclonal antibody (Ab50278, Abcam) at 1:1,000 dilution; LMP1 monoclonal antibody (Dako, Denmark) at 1:2,000 dilution. MCM-7 (Sigma-Aldrich) at 1:2,000 dilution was used as a loading control. Following washing with TBS-T, blots were incubated for 1 hour with the appropriate HRP-conjugated secondary antibody (Dako, Denmark). Proteins were visualised using the enhanced chemiluminescence (ECL) technique (GE healthcare, UK).

### 3.5. Immunohistochemistry

Immunohistochemistry was performed on normal tonsillar tissue removed at the time of tonsillectomy, and on 60 adult and 17 paediatric HL biopsies. Sections of paraffin-embedded tissues were cut at 4 µm thickness and dried at 60 °C for 1 hour. Sections were de-waxed, rehydrated and endogenous peroxidise activity blocked in 0.3% H_2_O_2_ (Sigma-Aldrich) for 15 minutes. Non-specific binding was blocked using 5× casein solution (Vector Laboratories, UK). Sections were incubated with the following primary antibodies for 16 hours at 4 °C: PRMT1 (sc59648; Santa Cruz); CARM1 (ab50278; Abcam) and PRMT5 (07-405; Upstate Millipore, CA, USA). Negative isotype controls were, mouse IgG1 (X0931; Alere, Chesire, UK), mouse IgG3 (MAB007; R & D Systems Europe Ltd, Abingdon, UK) and rabbit IgG (X0936; Alere), respectively. Dako Real Envision™ dual mouse/rabbit peroxide-conjugated was used as a secondary antibody. The staining was visualised with Dako Real Envision™ DAB chromagen and solution (Dako). Sections were counter-stained with Mayer’s Haematoxylin (Leica Microsystems, Peterborough, UK).

## 4. Conclusions

We have shown for the first time that EBV modulates the expression of those proteins involved in the regulation of arginine methylation. EBV infection of GC B cells, the presumptive progenitors of the Reed Sternberg cells found in HL, was followed by the up-regulation of the protein arginine methyltransferases CARM1, PRMT1 and PRMT5, and by the down-regulation of the deiminase, PADI4. This PRMT expression pattern was recapitulated in primary HL, and is entirely consistent with the results of gene expression profiling reported for micro-dissected HRS cells [27]. 

The up-regulation of PRMT1 within 96 hours of EBV infection is consistent with the time at which LMP1 can be first detected. However, up-regulation of PRMT5 and CARM1 was not seen until day 7, suggesting that their up-regulation is mediated either by other EBV genes or simply a consequence of proliferation. While we were also able to show that the proportion of cases with strong nuclear staining for PRMT1 was significantly greater in those who tested positive for EBV, it is important to note that PRMT1, PRMT5 and CARM1 were also over-expressed in EBV negative HL. Recently an inhibitor of arginine methyltransferases was shown to reduce Tax transactivation in HTLV-1 transformed cells while at the same reducing NF Kappa B activity [28]. As NF Kappa B is constitutively increased in both EBV positive and EBV negative HL, this would provide a common pathway explaining the up-regulation of PRMT1 in both virus positive and virus negative disease. 

Our observations offer some clues as to how EBV-induced changes in the expression of the PRMT might contribute to disturbances of B cell differentiation, and therefore to B cell lymphomagenesis. Signals processed through the B cell antigen receptor control both proliferation and differentiation. PRMT1 induced methylation of a conserved arginine residue in the CD79A subunit of the BCR has been shown to promote signals leading to B cell differentiation, an effect mediated in part by the modulation of calcium signalling [29]. We have shown that PRMT1 is up-regulated in GC B cells by LMP1 which has also been shown to increase the storage of Ca^2+^ in the endoplasmic reticulum in B cells [30]. Given that we have shown LMP1 can drive cells towards a post GC stage while at the same time hijacking the B cell transcriptional programme and subverting normal B cell differentiation [20], it will be important to investigate further the contribution of virus-induced de-regulation of PRMT1 to this process. 

More than thirty years ago inhibition of PRMT1 was first shown to inhibit Rous sarcoma virus induced chick embryo fibroblast transformation [31]. Small molecule inhibitors specific for the PRMT continue to be developed and while most of this activity has focused on inhibition of enzymatic activity, more recently compounds have been discovered which bind to PRMT substrates [32,33]. This continuing intensive effort provides a compelling reason for endeavouring to dissect the contribution to transformation of virus induced changes in the activity of these proteins.

## Appendix

**Supplemental Table 1:** Influence of histological type: comparison of immunohistochemical staining for PRMT1, CARM1 and PRMT5 in nodular sclerosis and mixed cellularity HL. Both nuclear and cytoplasmic PRMT expression was recorded as weak when staining of HRS cells was less than that observed in the surrounding lymphocytes; moderate when staining of HRS cells was as strong as that observed in the surrounding lymphocytes; and strong when staining of HRS cells was greater than that in the surrounding lymphocytes.

Influence of Histological Type: Comparison of Immunohistochemical Staining for PRMT1, CARM1 and PRMT5 in Nodular Sclerosis (NS, n = 40) and Mixed Cellularity (MC, n = 37)								
intensity of nuclear staining		PRMT1			CARM1		PRMT5	
		NS		MC	NS	MC	NS	MC
	strong	21		22	15	17	2	2
	moderate	17		15	13	13	7	5
	weak	1		0	9	2	7	6
	negative	1		0	1	3	23	24
		X^2^_ 3df_ = 3.0; p = 0.39			X^2^_ 3df_ = 5.5; p = 0.14		X^2^_ 3df_ = 0.4; p = 0.94	
intensity of cytoplasmic staining	strong	11	11		22	19	18	20
	moderate	17	18		12	12	19	17
	weak	5	2		4	3	1	0
	negative	6	6		0	1	1	0
		X^2^_ 3df_ = 1.3; p = 0.73			X^2^_ 3df_ = 1.8; p = 0.62		X^2^_ 3df_ = 3.1; p = 0.37	

**Supplemental Table 2:** Influence of age of diagnosis: comparison of immunohistochemical staining for PRMT1, CARM1 and PRMT5 in nodular sclerosis and mixed cellularity HL. Both nuclear and cytoplasmic PRMT expression was recorded as weak when staining of HRS cells was less than that observed in the surrounding lymphocytes; moderate when staining of HRS cells was as strong as that observed in the surrounding lymphocytes; and strong when staining of HRS cells was greater than that in the surrounding lymphocytes.

Influence of Age of Diagnosis in: Comparison of Immunohistochemical Staining for PRMT1, CARM1 and PRMT5 in Paediatric (n = 17) and Adult (n = 60) Cases							
intensity of nuclear staining		PRMT1		CARM1		PRMT5	
		adult	paediatric	adult	paediatric	adult	paediatric
	3	43	8	32	4	4	2
	2	32	8	26	9	12	5
	1	1	0	11	4	13	2
	0	1	0	4	0	47	7
		X^2^_3df_ = 1.1; p = 0.79		X^2^_3df_ = 4.0; p = 0.26		X^2^_3df_ = 3.7; p = 0.3	
intensity of cytoplasmic staining	3	22	4	41	9	38	7
	2	35	7	24	8	36	9
	1	7	0	7	0	1	0
	0	12	5	1	0	1	0
		X^2^_3df_ = 4.5; p = 0.21		X^2^_3df_ = 4.2; p = 0.24		X^2^_3df_ = 1.1; p = 0.78	
